# Whale shark *Rhincodon typus* foraging on small schooling fish in the northern Mexican Caribbean

**DOI:** 10.1111/jfb.70117

**Published:** 2025-06-16

**Authors:** Natalí Cárdenas‐Palomo, Emanuel Mimila‐Herrera, Iván Velázquez‐Abunader

**Affiliations:** ^1^ Pronatura Península de Yucatán, A. C Mérida México; ^2^ Departamento Recursos del Mar, Centro de Investigación y Estudios Avanzados del Instituto Politécnico Nacional Unidad Mérida Mérida México

**Keywords:** feeding ecology, filter feeding, lunge feeding, Mexican Caribbean, *Rhincodon typus*

## Abstract

The whale shark (*Rhincodon typus*) aggregates in the Mexican Caribbean from May to September to feed on mixed zooplankton patches and fish spawn. For the first time in this area, we present evidence of observations of whale sharks feeding on schools of small fish, two in 2017 (10 individuals per flight) and one in 2024 (19 individuals). This fact shows that there is another food source for the species at this site, which may be used as an alternative to meet their energy demands when zooplankton is scarce.

The whale shark (*Rhincodon typus*, Smith 1828) is the largest fish in the world, reaching lengths of up to 20 m (Colman, 1997). The species has been listed as endangered on the Red List of the International Union for Conservation of Nature (IUCN) since 2016 (Pierce & Norman, 2016) and is one of three filter‐feeding sharks found in the oceans worldwide (Compagno, 1984).

Whale sharks are known to feed primarily on zooplankton throughout the world. Some of their major prey groups include copepods, fish eggs, euphausiid krill, sergestids, crab larvae and chaetognaths (Boldrocchi et al., 2020; Cárdenas‐Palomo et al., 2015; Heyman et al., 2001; Motta et al., 2010; Robinson et al., 2013; Rohner et al., 2015; Rowat & Brooks, 2012). Gudger (1941) first proposed that whale sharks might consume small fish, based on observations by colleagues in the Seychelles, Cuba, the Bahamas and the southeastern United States, although no conclusive evidence was available at the time. More recent studies (Afonso et al., 2014; Boldrocchi & Bettinetti, 2019) suggest that whale sharks also feed on fish, using behaviours that are not yet fully understood (Montero‐Quintana et al., 2021).

Due to its size, the whale shark must consume a large amount of food per day. Motta et al. (2010) estimated that a whale shark of 6 m in length could consume 2.76 kg of plankton per hour, which equates to 20.7 kg of plankton per day (assuming that it spends ~7.5 h per day feeding). The whale shark has a circumtropical distribution, inhabiting only tropical and subtropical warm waters (Rowat & Brooks, 2012). These regions have the lowest plankton productivity, with high spatio‐temporal variation in the distribution of plankton patches (Lalli & Parsons, 1997). For this reason, the whale shark must feed very efficiently, using a variety of strategies to find sufficient food in these relatively unproductive waters (Rohner & Prebble, 2021). Approximately 30 whale shark ‘constellations’, a term coined to refer to whale shark aggregations (Dove, 2021), have been identified worldwide as hotspots for the species; in most cases, these are sites where mostly juvenile males aggregate to feed (Norman et al., 2017). From May to September, large numbers of whale sharks aggregate in the marine area located at the north of the Mexican Caribbean, making it one of the most important sites for the species worldwide (de la Parra Venegas et al., 2011; Trujillo‐Córdova et al., 2020). This site includes four national natural protected areas, two of which are the ones where the highest number of whale shark sightings have been recorded: the Whale Shark Biosphere Reserve and a zone called the Azul, which is within the Mexican Caribbean Biosphere Reserve (Figure S1). The Whale Shark Biosphere Reserve has high zooplankton biomass due to the Yucatan upwelling (Merino, 1997), whereas the Azul zone has dense masses of fish eggs (Cárdenas‐Palomo et al., 2015; Motta et al., 2010). In this brief communication, we present the first evidence of whale sharks feeding on a school of small fish in the Mexican Caribbean.

As part of the activities of the Whale Shark Sustainable Management Programme in the Mexican Caribbean, implemented by Pronatura Península de Yucatán and Cinvestav Mérida, aerial surveys have been conducted once a month from May to September since 2016 to count and record the geographic location of whale sharks. Surveys were conducted using a single‐engine high‐wing aircraft Cessna C206. The average height of the overflights was 367 m, and the average speed was 165 km h^−1^. The flight route, which lasted ~2.5 h, included the aggregation area of the species known as the Azul zone and part of the Whale Shark Biosphere Reserve.

In addition, marine surveys were conducted using outboard motorboats to collect whale shark sightings and environmental data. During these surveys, a surface zooplankton trawl was conducted for 5 min using a 300‐μm mesh plankton net with an opening diameter of 0·3 m, following the recommendations of the Cooperative Investigations of the Caribbean and Adjacent Regions (UNESCO, 1979). Zooplankton biomass was determined using the wet weight technique and expressed in milligram per metre cube (Omori & Ikeda, 1984) as a proxy for food availability to the whale shark.

In 77 aerial and marine surveys conducted by the Whale Shark Sustainable Management Programme from 2005 to 2015, no sightings of sharks feeding on schools of fish had been recorded. During 44 overflights conducted by this programme since 2016, we have observed this feeding behaviour only on three occasions, which we report here. Due to the limitations of aerial observation, it was not possible to identify the species of fish targeted by the whale shark. However, we infer that the school probably consisted of a sardine species. A previous study of the fish community on Contoy Island, which is located close to the site where whale sharks were observed feeding, reported that the most common species of small pelagic fish was the scaled sardine (*Harengula jaguana*, Poey 1865) (Vega‐Cendejas & Hernández‐de‐Santillana, 2014). Although tour operators and local fishermen have reported sightings of whale sharks feeding on sardines, this is the first published report for this area with photographic evidence. The three events of whale sharks feeding on schools of fish were recorded on 18 July and 19 September 2017 and 5 September 2024 (Figure 1). These events occurred within the Whale Shark Biosphere Reserve, a protected area where the species commonly feeds on zooplankton, consisting mainly of sergestids, calanoid copepods, chaetognaths, appendicularia and fish larvae (Cárdenas‐Palomo et al., 2010; Motta et al., 2010).

**FIGURE 1:**
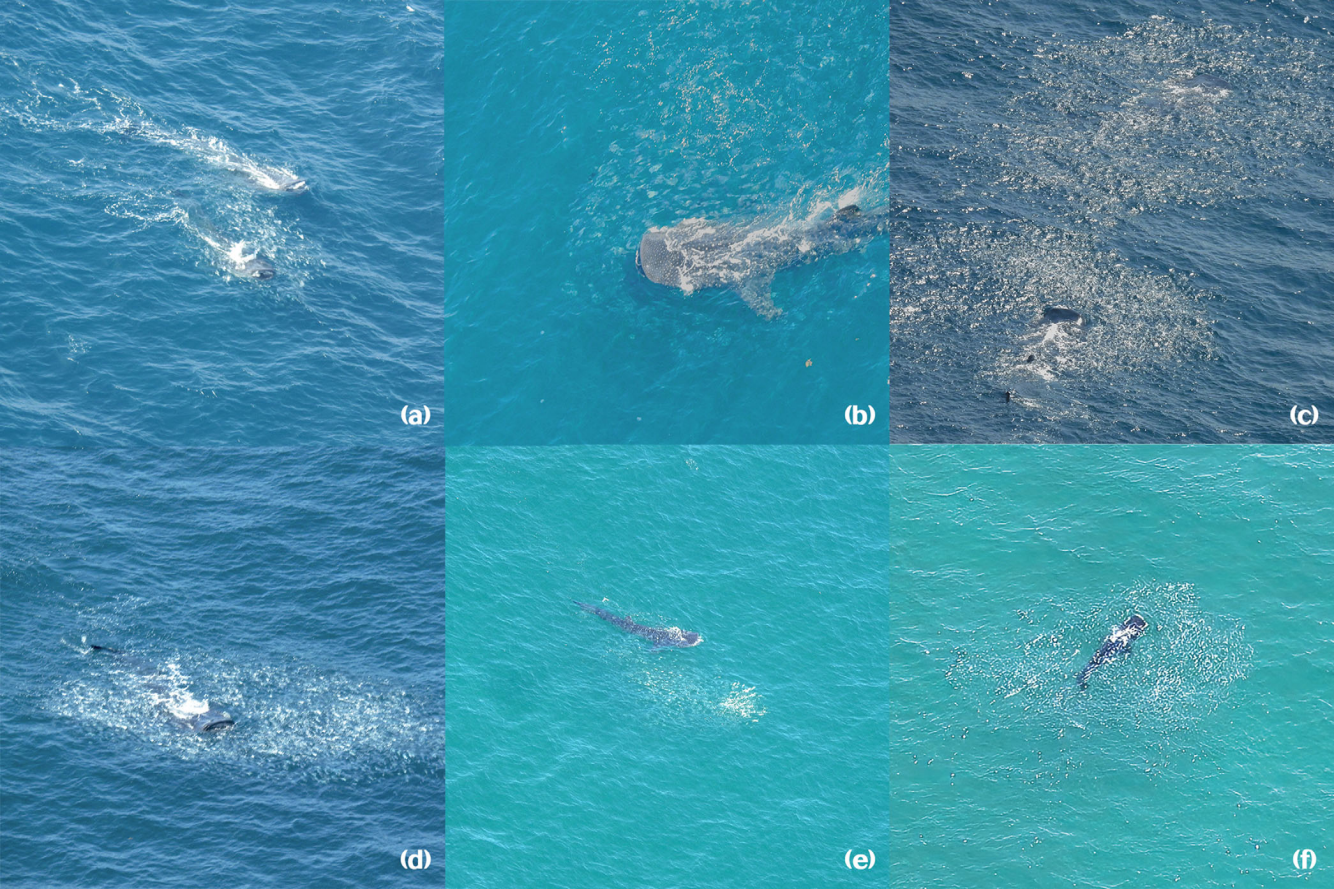
Whale sharks feeding on small schools of fish in the northern Mexican Caribbean. Aerial images taken on (a) 18 July 2017, (b–d) 19 September 2017 and (e, f) 5 September 2024.

During the July 2017 event, eight sharks were observed feeding on small fish. We observed two whale sharks simultaneously attacking the school of fish and performing individual lunges, with one whale shark observed lunging towards the school of fish. During the feeding event in September 2017, we first observed two whale sharks lunging towards the school of fish one after the other, followed by a single lunging session in which only one whale shark carried out lunging behaviours. At the second site, we observed one whale shark lunging towards the school, followed by two sharks lunging towards the school at the same time. In September 2024, nine whale sharks were observed feeding on small fish at two sites close to each other. At the first site, two individuals fed on the same school of fish, and at the second site, seven whale sharks fed on two schools of fish.

Lunge feeding is a whale shark strategy that has been described recently. During lunge feeding, the whale shark accelerates towards schools of small fish with its mouth open and strong movements of its caudal fin (Montero‐Quintana et al., 2021). The observed behaviour of whale sharks feeding on schools of small fish in the Mexican Caribbean during 2017 and 2024 was consistent with the description of this feeding strategy.

Whale sharks in the Mexican Caribbean exhibit three main feeding behaviours: (1) passive feeding or ram feeding, in which they swim at low speed with their mouth partially or fully open at the surface or at depth, often when prey density is low (Nelson & Eckert, 2007) or when prey is small or immobile, such as fish eggs (Rohner & Prebble, 2021); (2) active feeding or suction filter feeding, which involves rapid mouth opening and gill expansion to ingest water, typically when plankton densities are high or plankton are mobile, such as small fish and sergestids (Nelson & Eckert, 2007); and (3) stationary feeding or vertical feeding, in which sharks feed while stationary by actively sucking water, often when prey is aggregated in patches (Heyman et al., 2001) or when feeding on small fish (Boldrocchi & Bettinetti, 2019; Gudger, 1941).

A feeding behaviour known as yo‐yo feeding has been reported for whale sharks (Rohner et al., 2015), in which they feed by active suction as they approach the surface after oscillatory bounces from dives of ~10 m. This behaviour has been reported on Mafia Island, where sharks feed on sergestids, but has not been reported in the Mexican Caribbean. On the contrary, Whitehead and Gayford (2023) report the first observation of bottom feeding in whale sharks in Baja, California Sur México. D'Antonio et al. (2024) also report seafloor gulping at Ningaloo Reef, Australia, which may be associated with foraging behaviour. Although these behaviours have not been reported in the Mexican Caribbean, fatty acid analysis has suggested that whale sharks may also feed on demersal zooplankton as a complementary food source (Cárdenas‐Palomo et al., 2018).

During flights conducted in July 2017 and September 2024, whale sharks were also reported in the Azul zone (two and three individuals, respectively), with no evidence to suggest they were feeding. Since 2009, the Azul is the area where the most sightings of the species have been recorded. The mean zooplankton biomass in the Azul zone (>1500 mg m^−3^) is significantly higher than the zooplankton biomass reported within the Whale Shark Biosphere Reserve (~300 mg m^−3^). In addition, the whale shark groups observed in the Azul zone tend to be more numerous than those observed within the Whale Shark Biosphere Reserve (Trujillo‐Córdova et al., 2020). Therefore, the observation of only five organisms in the Azul zone and more individuals inside the Reserve in September 2024 may indicate that spawning biomass was low or absent.

Zooplankton biomass data are not available from the specific days when whale sharks were observed feeding on schools of fish, but samples were collected at other times during the same season. The average zooplankton biomass in the Azul zone was 16,600 and 5450 mg m^−3^ in July 2017 and June 2024, respectively. In samples collected in September 2017, the average biomass in the Azul zone was 9100 mg m^−3^, and in a sample collected on 12 September 2024, the biomass value was 1022 mg m^−3^ (Cárdenas‐Palomo et al., 2025). These data show that in both years the biomass of zooplankton decreased over the course of the season, with the lowest values in September.

Most observations of whale sharks feeding on schools of small fish were recorded in September, at the end of the whale shark season and when zooplankton biomass in the Azul zone is expected to decline, which may encourage the sharks to utilize alternative food sources. Schools of small fish are highly attractive to various predators in the marine environment (sharks, dolphins, sailfish, etc.), as they are dense patches of energy‐rich prey. The whale shark constellation located in the Mexican Caribbean is an important feeding site for the species, where organisms recharge and continue their migration route (Green & Giese, 2004).

The whale shark is known to be an opportunistic species that can feed on a wide variety of prey (Colman, 1997). Decades ago, it was reported that whale sharks had been observed near schools of sardines and anchovies; at the time, it was suggested that they were not feeding on them but rather that they co‐occurred due to high plankton densities (Gudger, 1941; Nelson & Eckert, 2007). However, recent publications have reported evidence of whale sharks feeding on schools of small fish at other aggregation sites. Boldrocchi and Bettinetti (2019) reported seven juvenile whale sharks feeding on a school of baitfish in Djibouti; Montero‐Quintana et al. (2021) provide evidence of whale sharks feeding on schools of anchovies (*Anchoa* sp.) on four occasions in two consecutive years in Bahia de Los Angeles, Baja California, Mexico; and Lester et al. (2022) provide evidence of whale sharks feeding on bait balls in the presence of other predators in Western Australia.

The flexible feeding strategies of whale sharks may maximize their energy availability in tropical environments where plankton is dispersed, and productivity is low. Although the feeding behaviour of whale sharks in schools of fish needs to be studied in more detail, it is possible that it is helping the species to adapt to its changing habitat. Recent publications and this brief communication suggest that schools of small fish may be a more important food source than previously thought for this typically planktophagous species.

## AUTHOR CONTRIBUTIONS

Natalí Cárdenas‐Palomo was involved in conceptualization and writing of the original draft, and review and editing; Emanuel Mimila‐Herrera contributed to fieldwork and writing, and review and editing; Iván Velázquez‐Abunader contributed to the drafting of the manuscript. All authors gave final approval for publication.

## CONFLICT OF INTEREST STATEMENT

All authors declare that they have no conflict of interest.

## Supporting information


**Figure S1.** Location of the whale shark aggregation site in the northern Mexican Caribbean.
