# Supplementary figures and images for: Whale shark Rhincodon typus foraging on small schooling fish in the northern Mexican Caribbean
Source: J Fish Biol. 2025 Jun 16;107(4):1436–40. doi: 10.1111/jfb.70117 (PMC12536054; doi:10.1111/jfb.70117)

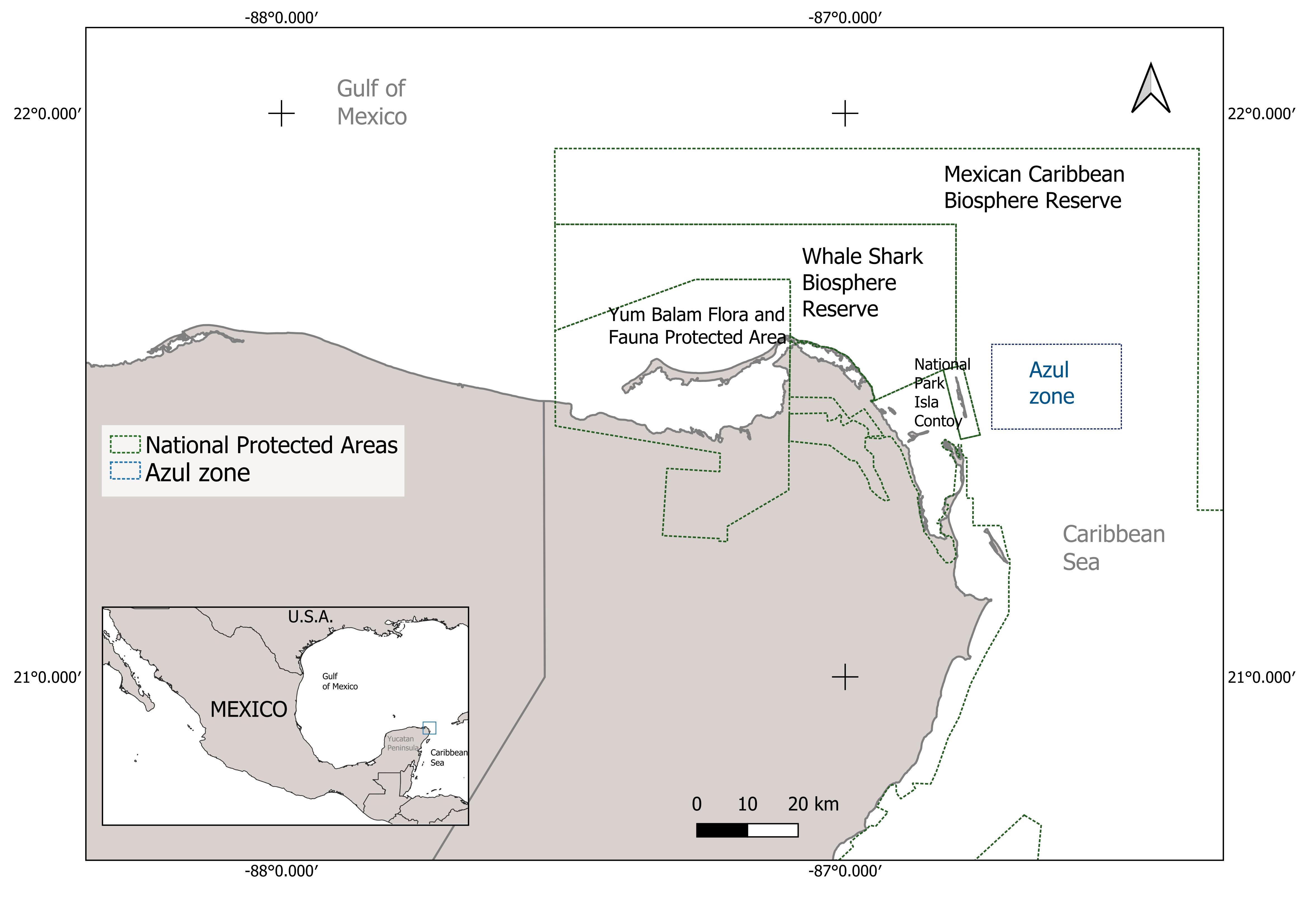

Supplement: Supplementary file 1 — Figure S1. Location of the whale shark aggregation site in the northern Mexican Caribbean. [file JFB-107-1436-s001.jpeg]
